# Enzymatic Bioprospecting of Fungi Isolated from a Tropical Rainforest in Mexico

**DOI:** 10.3390/jof8010022

**Published:** 2021-12-28

**Authors:** Karla Peraza-Jiménez, Susana De la Rosa-García, José Javier Huijara-Vasconselos, Manuela Reyes-Estebanez, Sergio Gómez-Cornelio

**Affiliations:** 1 Laboratorio de Microbiología Aplicada, División Académica de Ciencias Biológicas, Universidad Juárez Autónoma de Tabasco, Villahermosa 86150, Mexico; karla.peraza@ujat.mx (K.P.-J.); susana.delarosa@ujat.mx (S.D.l.R.-G.); 2 División Académica de Ciencias Agropecuarias, Universidad Juárez Autónoma de Tabasco, Villahermosa 86298, Mexico; javier.huijara@ujat.mx; 3 Laboratorio de Microbiología Ambiental y Biotecnologia, Universidad Autónoma de Campeche, Campeche 24039, Mexico; mamreyes@uacam.mx; 4 Ingeniería en Biotecnología, Universidad Politécnica del Centro, Villahermosa 86290, Mexico

**Keywords:** hydrolases, oxidoreductases, enzyme extracellular, screening

## Abstract

The humid tropical environment provides an ideal place for developing a high diversity of plants; this is why it is an interesting site for the enzymatic bioprospecting of fungi that are responsible for the recycling of organic matter in an efficient and accelerated way and whose enzymes could have multiple biotechnological applications. For this study, 1250 isolates of macroscopic and microscopic fungal morphotypes were collected from soil, leaf litter, and wood. One hundred and fifty strains (50 from each source) were selected for the enzymatic screening. From the first phase, 51 strains with positive activity for laccase, protease, amylase, xylanase, and lipase enzymes were evaluated, of which 20 were isolated from leaf litter, 18 from the soil, and 13 from wood. The 10 best strains were selected for the enzymatic quantification, considering the potency index and the production of at least two enzymes. High laccase activity was detected for *Trametes villosa* FE35 and *Marasmius* sp. CE25 (1179 and 710.66 U/mg, respectively), while *Daedalea flavida* PE47 showed laccase (521.85 U/mg) and protease activities (80.66 U/mg). *Fusarium* spp. PH79 and FS400 strains had amylase (14.0 U/mg, 49.23 U/mg) and xylanase activities (40.05 U/mg, 36.03 U/mg) respectively. These results confirm the enzymatic potential of fungi that inhabit little-explored tropical rainforests with applications in industry.

## 1. Introduction

The geographical position of Mexico (Nearctic and Neotropical) favors a mountainous tropical climate with a great variety of ecosystems and diversity of species, occupying fifth place within the group of megadiverse countries in the world, hosting around 12% of the planet’s biodiversity [1,2]. The humid tropics of Mexico are characterized by typical high jungle perennial vegetation and environmental conditions with high temperatures and annual rainfall, which generates an accelerated and continuous environment in the recycling of nutrients, decomposition of organic matter, and competition for substrates [3,4]. All of these peculiarities make these environments optimal for developing a high diversity of fungi; therefore, in this area, it is possible to find fungi that produce hydrolytic and lignin-degrading enzymes with biotechnological potential that are still unexplored [5].

The fungi’s physiological adaptability and multienzyme metabolic system constitute the base of their amazing ability to develop under diverse environmental conditions, considered the drivers of natural restoration of the ecosystems. They are natural decomposers of organic matter to absorb their nutrients, thus allowing recycling, mineralization, and compound release for the community and the ecosystem; therefore, fungi play a vital role in the global carbon cycle [6,7].

Extracellular enzymes of fungal origin, both oxidoreductive and hydrolytic, have been reported for various industrial and biotechnological applications, like medicine [8], agriculture [9], pulp and paper [10], textiles [11], detergents [12], food processing [13], and biofuels industries [14]; as well as bioremediation [15]. In addition, fungal enzymes have a more significant advantage over those derived from plants or animals due to their easy handling, rapid production in low-cost media, higher yields, and catalytic activity [16].

Although the tropics have the most remarkable diversity of fungal species, only a few enzymatic bioprospecting studies have reported on the Mexican southeast [17,18,19], generally focused on lignocellulolytic enzyme activity. Therefore, this work aims to explore and evaluate, qualitatively and quantitatively, five enzymes secreted by fungi isolated from three sources (litter, soil, and wood) collected from the tropical rainforest.

## 2. Materials and Methods

### 2.1. Biological Sampling

The samples were collected from three conserved tropical sites in the sierra region of the south of Mexico: Coconá (17°33′46.6″ N, 92°55′31″ W); Puyacatengo (17°31′34.2″ N, 92°55′31″ W) and La Florida biological station (17°27.8′33″ N, 99°45′99″ W). It is a mountainous area of tropical jungle with water bodies like rivers and streams where a humid climate prevails with rains all year round. The region registers the highest rainfall in Mexico, with an annual average between 2214 and 3382 mm, with two different seasons: rainy season (from August to March), dry season (from April to July). The annual average temperature is between 22 and 26.8 °C.

### 2.2. Isolation of the Fungi

The fungi were isolated in potato dextrose agar (PDA) with chloramphenicol (15 µg/mL) to reduce bacterial growth. Soil fungi were isolated by washing and filtrating particles techniques using microsieves reported by [20]; after removing the moisture excess, five soil particles were inoculated on each plate. The fungal strains obtained from leaf litter and sporocarps on decaying wood were superficially washed with 2% sodium hypochlorite for 1 min, 70% ethanol for 1 min, and three washes were performed with sterile distilled water [21]. The sporocarps (MAF) were cut into small fragments and inoculated with a fine needle, while the leaf litter was cut into fragments of 1 cm^2^, and five fragments were inoculated in the plate. The plates were incubated at 28 °C, and periodic checks were carried out every third day for one month. Emerging fungi were transferred onto new plates with PDA until obtaining pure cultures. 

### 2.3. Morphological Identification

1250 macroscopic (MAF) and microscopic fungal (MIF) were isolated from soil, leaf litter, and wood. The strains were grouped by source, morphotype, and percentage of occurrence (data not shown); later, 50 strains from each source were selected (soil, leaf litter, and wood), excluding the typical morphologies of *Penicillium*, *Cladosporium*, and *Trichoderma*. The fungal isolates were identified according to macroscopic and microscopic characteristics—such as mycelia, fruiting bodies, arrangement of conidia, among others—using taxonomic keys and consulting specialized references [22,23,24,25,26,27,28]. 

The MIF isolates from leaf litter and soil that did not show spores were inoculated in corn agar, oatmeal agar, potato carrot agar, V8 agar, humic acid agar, and leaf litter agar to promote sporulation, incubating them for six weeks under continuous black light at 28 °C [20]. All isolates were maintained in PDA slant tubes and stored in 20% glycerol at −80 °C, and mycelium plugs were stored in sterile distilled water at room temperature (28–30 °C).

### 2.4. Qualitative Assay in Solid Medium

For the enzyme screening of the 150 selected strains, plates with minimal mineral medium (0.6 g/L NH_4_NO_3_, 0.2 g/L KH_2_PO_4_, 0.2 g/L MgSO_4_·7H_2_O, 0.001 g/L FeSO_4_·7H_2_O, 15 g/L bacteriological agar) were supplemented with substrates specific: 0.5 mM 2,2′-azino-bis 3-ethylbenthiazoline-6-sulfonic acid (ABTS) and 0.9 mM guaiacol for laccases [29,30], 6% (*v*/*v*) olive oil, 0.2% (*v*/*v*) Tween 80, and 0.001% rhodamine B solution, at pH 7.0 for lipases [31], 1% soluble starch, at pH 6.0 for amylases [32], 1% birch xylan for xylanases [33], and 1% skim milk powder for proteases [34]. All the media were sterilized for 15 min at 120 °C. An inoculum of the fungus (0.5 cm^2^) was placed in the center of the Petri dishes and incubated at 28 °C for 15 days. All strains that showed a blue-green oxidation halo for laccases were considered positive, as well as a clear area around the fungal growth product of the hydrolysis of starch, xylan, and casein, while an orange fluorescence halo was observed for lipase activity. All the tests were carried out in triplicate. The activity was reported as potency index (PI) measured every 24 h, as the halo diameter formed between the mycelial growth’s diameter [35].

### 2.5. Basal Medium for Enzyme Production

To evaluate laccase activity, the modified medium of Sivakumar et al. [36], was used (10 g/L fructose, 2.5 g/L malt extract, 2.5 g/L yeast extract, 1 g/L KH_2_PO_4_, 0.05 g/L (NH_4_)_2_SO_4_, 0.5 g/L MgSO_4_, 0.01 g/L CaCl_2_, 0.001 g/L MnSO_4_, 0.001 g/L ZnSO_4_, and 0.2 mM CuSO_4_·5H_2_O, adjusted at pH 4.8). For amylase and xylanase activity, we used 2.5 g/L yeast extract, 0.6 g/L NH_4_NO_3_, 0.2 g/L KH_2_PO_4_, 0.2 g/L MgSO_4_·7H_2_O, 0.001 g/L FeSO_4_·7H_2_O supplement 10 g/L soluble starch for amylase and 10 g/L birch xylan, 5 g/L wheat bran for xylanase, both media were adjusted to pH 6.0. To evaluate protease and lipase activity the following medium was used: 2.0 g/L yeast extract, 0.02 g/L MgSO_4_, 2.0 g/L glucose, and 0.1 g/L KH_2_PO_4_, supplemented with 20 g/L casein and 2% *v*/*v* olive oil, respectively.

### 2.6. Enzyme Production by Submerged Fermentation

The submerged fermentation medium for enzyme production was sterilized for 20 min at 120 °C; 100 mL of the sterile basal medium was prepared as earlier described with the appropriate carbon source in 250 mL flasks. Each fungus was grown from two to seven days on PDA, and 8 mm agar plugs, then were cut from the agar and transferred in flasks, keeping them in an orbital shaking at 140 rpm at 28 °C for 16 days. 

The enzyme quantification was performed by protein content according to the Lowry method [37]. For this purpose, 5 mL of the supernatant were taken every second day, centrifuging at 1487× *g* for 10 min, followed by filtration on Whatman filter paper number 1. The experiments were performed in triplicate for each fungal strain.

#### 2.6.1. Quantitative Assay Laccase Activity

Laccase (lignin peroxidase) activity was determined by oxidation of the ABTS. The reaction mixture contained 0.5 mM ABTS, 0.1 M sodium acetate buffer (pH 4.5), and 100 µL of supernatant. Substrate oxidation was monitored by increasing absorbance at 420 nm (ε420 = 3.6 × 10^4^ M^−1^ cm^−1^) for 5 min. The enzymatic activity was expressed as U = 1 µmol of ABTS oxidized per min at 25 °C [29].

#### 2.6.2. Quantitative Assay Amylase Activity

Amylase activity was measured with the DNS method (3,5-dinitrosalicylic acid) [38] by the reducing sugars released during the assays, using 1% soluble starch as substrate dissolved in 0.1 M sodium citrate buffer, at pH 5.6. The reaction mixture comprised 0.5 mL of the enzyme supernatant and 0.5 mL of pH buffer followed by incubation in a water bath at 40 °C for 30 min. The reaction was stopped with 2 mL of the DNS reagent followed by heating to 100 °C for 5 min. Optical density was read at 540 nm against a blank without the enzyme, and a glucose standard curve (0.03125–2 mg/mL, y = 0.9783x + 0.0393, R^2^ = 0.9994) was performed under the same assay conditions. Amylase activity was expressed in U/mL defined as the amount of enzyme that releases 1 µmol of reducing sugar equivalent to glucose per mL in one minute [39].

#### 2.6.3. Quantitative Assay Xylanase Activity

Xylanase activity was evaluated by the DNS method [38] to quantify the reducing sugars formed during the hydrolysis of the endo-1,4 β-D bonds of the xylan skeleton. The reaction mixture consisting of 0.5 mL of the culture supernatant in 0.05 M of sodium acetate buffer, pH 5.3, and 0.5 mL of 1% of birch xylan followed by incubation at 40 °C for 30 min. The reaction was stopped by adding 2 mL of the DNS reagent; then, samples were heated at 80 °C for 5 min, the absorbance was read at 540 nm. A xylose standard curve (0.3125–2 mg/mL, y = 0.488x + 0.0297, R^2^ = 0.9997) was performed under the same test conditions. Xylanase activity was expressed as the amount of enzyme that releases 1 µmol equivalent of xylose per mL in one minute [40].

#### 2.6.4. Quantitative Assay Protease Activity

Protease activity was quantified using the technique proposed by Cupp-Enyard et al. [41]. For this test, 0.65% of a casein solution was prepared in 50 mM potassium phosphate buffer, pH 7.5, 5 mL of the solution were added in test tubes and incubated in a water bath at 37 °C for 5 min. After incubation, 1 mL of the enzyme supernatant was added to the test tubes and vortexed for 10 min. The same procedure was carried out for the blank without the supernatant. The reaction was stopped with 5 mL of 110 mM trichloroacetic acid. Afterward, the samples were filtered, and 2 mL of the filtrate was added to 5 mL of 500 mM sodium carbonate and 1 mL of the 0.5 mM Folin’s reagent; all of this was mixed perfectly and incubated at room temperature in the dark for 30 min. The absorbance was measured at 660 nm, and a tyrosine standard curve (1.56–50 µg/mL, y = 0.00121x + 0.0238, R^2^ = 0.9905) was developed under the same assay conditions. One unit of protease was defined as the amount of enzyme that releases 1 µmol of tyrosine per minute under these conditions [34].

#### 2.6.5. Quantitative Assay Lipase Activity

Lipase activity was quantified using ρ-nitrophenyl palmitate (ρ-NPP) as substrate, dissolved in 10% isopropanol (10 mL) and mixed with 50 mM potassium phosphate buffer (90 mL) at pH 8.0, 207 mg of sodium deoxycholate, and 100 mg of gum arabic for a final concentration of 790 µM of the ρ-NPP substrate solution, finally, 50 µL of Triton X-100 were added for clarification. For the test, 2.4 mL of the substrate solution were incubated with 0.1 mL of the enzyme supernatant at 37 °C for 15 min. The samples were measured in a spectrophotometer at 410 nm with a standard curve for ρ-nitrophenol (0.33–27 µg/mL, y = 0.0173x + 0.0003, R^2^ = 0.9994). The enzymatic activity was expressed as U = 1 µmol of ρ-nitrophenol released per mL in one minute [42,43].

Total protein concentration was determined using the method of [37] with a calibration curve (0.0039–2 mg/mL, y = 1.477x − 0.083, R^2^ = 0.9879) with bovine serum albumin as a protein standard.

### 2.7. Statistical Analysis

All enzyme quantification was performed in triplicate, and results were analyzed with multiple comparisons of means, performing a one-way analysis of variance (ANOVA), followed by a Tukey test. A significance of α = 0.05 was considered in the analyses with the statistical package Stagraphics 7.

## 3. Results

From the 1250 fungi isolates of the humid tropical rainforest in Mexico, 150 (50 from soil, 50 from leaf litter, and 50 from sporocarps of wood) were selected and evaluated to determine their enzymatic potential. Regarding the taxonomic analysis, of the 51 active strains, 5 were identified at the species level, 32 at the genus level, 4 at the family level, and 10 as mycelia sterilia. The composition of the fungal strains selected for each source was analyzed, finding *Auricularia*, *Trametes*, *Neonothopanus*, *Hexagonia*, *Marasmius*, *Marasmiellus*, *Daedalea*, *Panus, Rigidoporus* genera, and two Basidiomycetes and one Ascomycete unidentified on decaying wood trunks. Regarding the leaf litter isolates, *Acremonium*, *Fusarium*, *Aspergillus*, *Nodulisporium*, *Drechslera*, *Monodictys* genera, a member of the Xylariaceae family, and eight mycelia sterilia were identified. In contrast, from the soil, *Acremonium*, *Cylindrocarpon*, *Graphium*, *Gliomastix*, *Paecilomyces* genera, a Xylariaceae family, and two mycelia sterilia—besides *Aspergillus* and *Fusarium* genera—were found (Table 1).

From the 150 fungal strains evaluated, 51 strains (13 from wood, 20 from leaf litter, and 18 from the soil) showed positive results in the qualitative plate method evidencing the presence of extracellular enzyme oxidoreductases (laccases) and hydrolases (amylase, lipase, proteases, and xylanases).

**Figure 1:**
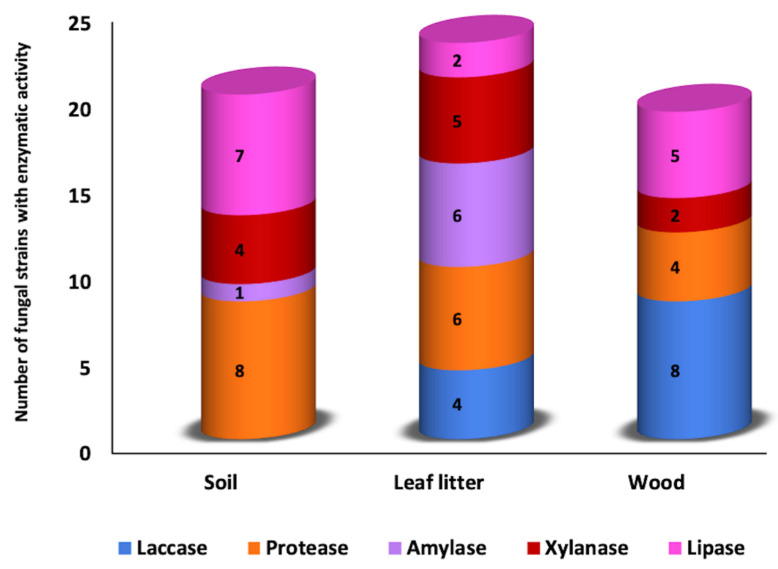
Distribution of the enzymatic activity of fungal strains and their relationship with isolation substrates from a tropical forest.

A close relationship between the enzymatic activity and the isolation source was observed; 8% of the evaluated strains showed laccase activity, all isolated from wood and leaf litter. For hydrolytic enzymes, 4.6% expressed amylase activity (leaf litter and soil), while 9.3% of the strains evaluated showed lipase activity, 12% for proteases, and 7.2% for xylanases of the three sources (Table 1 and Figure 1). The collection sites were not significantly different (*p* < 0.05) regarding the number of strains and enzymatic activity of the fungi isolated on the three sources evaluated.

### 3.1. Qualitative Plate Test

**Figure 2:**
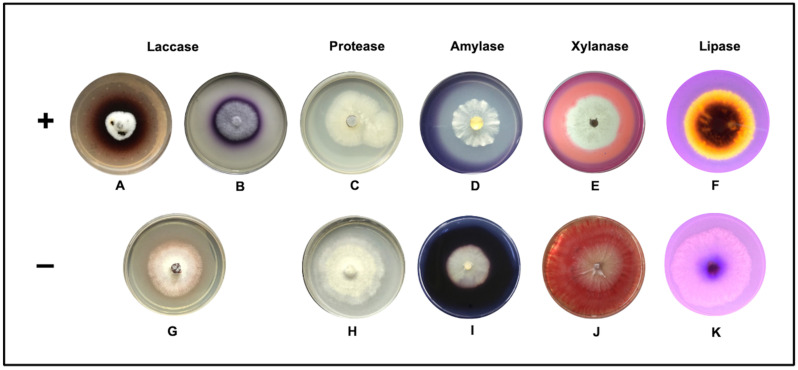
Qualitative enzymatic assay on solid medium with visible enzymatic activity (**A**–**F**) and without activity (**G**–**K**).

The laccase activity was detected by the oxidation of the ABTS (blue-green halo) and guaiacol (brown halo) around the colony, mainly of fungi known as lignin degraders or white rot (Figure 2A,B), the strains with the highest activity were *Trametes villosa* FE35, *Daedalea flavida* PE47 *Panus* sp. PE43, *Rigidoporus* sp. FE55, and *Marasmius* sp. (CE25), with enzymatic potency index (PI) of 2.3, 2.2, 2.1, 1.9, and 1.7, respectively, recovered from wood (Table 1). Protease activity was evident with a clear zone (substrate hydrolyzed) around the colony (Figure 2C) in isolated strains of wood, Basidiomycete CE34 (3.0), CE10 (2.3), and *Daedalea flavida* PE47 (2.4) and *Monodictys* sp. CH616 (2.6) recovered from leaves. The best activity was registered for the recovered fungi from soil as Ascomycete PS1130 (3.2), *Fusarium* sp. FS400 (3.0), *Acremonium* sp. PS595 (2.8), *Aspergillus* sp. PS948 (2.7), *Gliomastix* sp. PS1298 (2.4), and *Paecilomyces* sp. FS446 (2.3).

Regarding the detection of amylase, positive results were visualized after adding an iodine solution (0.3% iodine and 0.6% potassium iodide) that caused a clear zone around the colonies because of the hydrolysis of starch (Figure 2D). The amylase activity was not detected for strains recovered from wood. In isolated strains of leaf litter (PI), they stand out *Aspergillus* sp. CH693 (2.5), the mycelia sterilia FH175 (2.4) and FH338 (2.2), *Nodulisporium* sp. PH225 (1.8), *Fusarium* sp. PH79 (1.9), and Xylariaceae PH208 (1.9), while in the soil, only *Fusarium* sp. FS720 (2.5).

In xylanases, the visualization was by adding a Congo red solution (0.4%) that stains the plates red, observing a lighter area around the colonies that indicate the presence of xylanase activity (Figure 2E). The activity was recorded in the three sources, in leaf litter was observed for *Aspergillus* sp. CH665 (3.5), *Fusarium* spp. FH702 (3.2), and PH79 (1.8), *mycelia* sterilia PH706 (2.6), Xylariaceae PH208 (1.9), in wood *Rigidoporus* sp. FE55 (1.8) and *Neonothopanus* sp. FE33 (1.6); in soil *Fusarium* sp. FS720 (1.7), *Cylindrocarpon* sp. FS45 (1.6), Xylariaceae CS708 (1.4), and *Acremonium* sp. CS330 (1.2) (Table 1).

Regarding lipase activity, the positive strains were determined by forming an orange-fluorescent halo around the colonies visible under UV light at 350 nm, a product of the interaction of rhodamine B with the fatty acids released during the enzymatic hydrolysis of the triacylglycerols (Figure 2F). The lipases were positive for the fungi isolated from the three sources: in the soil, *Graphium* spp. FS835 (2.3) and PS1143 (2.2), two mycelia sterilia FS943 (2.3) and FS907 (2.2), *Fusarium* spp. FS903 (2.2) and FS400 (2.1), *Aspergillus* sp. CS1022 (2.2); in leaf litter to *Aspergillus* sp. CH693 (2.3) and *Fusarium* sp. FH676 (2.3); and in wood *Auricularia* sp. FE15 (2.5), *Marasmius* spp. CE25 (2,3) and PE38 (2,2), *Daedalea flavida* PE47 (2,2) and *Trametes villosa* FE35 (2,1), (Table 1).

### 3.2. Enzymatic Activity Quantification

**Figure 3:**
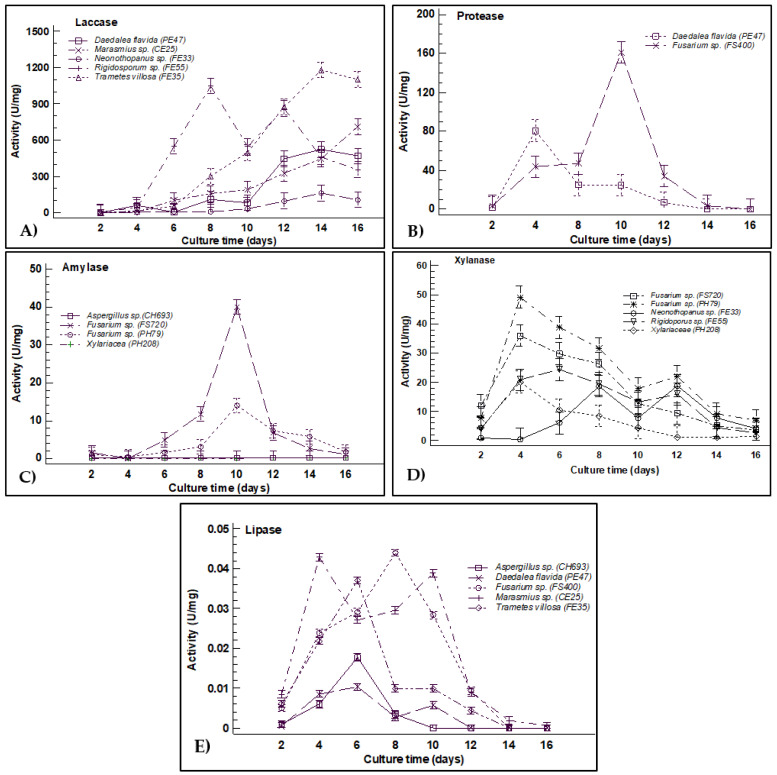
Enzymatic activity quantification of the ten selected fungi expressed in U/mg: (**A**) Laccase, (**B**) Protease, (**C**) Amylase, (**D**) Xylanase, and (**E**) Lipase.

In a second test, the enzyme expression was quantified by liquid fermentation of the 10 fungi that showed the highest PI and more than one enzyme in the qualitative tests. These were *Marasmius* sp. CE25, *Neonothopanus* sp. FE33, *Trametes villosa* FE35, *Daedalea flavida* PE47, *Aspergillus* sp. CH693, *Rigidoporus* sp. FH55, Xylariaceae PH208, and three *Fusarium* spp. FH79, FS400, and FS720. Enzymatic quantification of blue multi-copper oxygen oxidoreductases was performed for the wood isolates of *Trametes villosa* FE35, *Daedalea flavida* PE47, *Marasmius* sp. CE25, *Neonothopanus* sp. FE33, and *Rigidoporus* sp. FH55. The results of this quantification show that these fungi reach maximum activity between 14 and 16 days of culture except for *Rigidoporus* sp. FH55, which was eight days later. The highest activity was for *Trametes villosa* FE35 with 1179 U/mg (82.78 U/mL), followed by *Rigidoporus* sp. FH55 with 1044 U/mg (73.06 U/mL), *Marasmius* sp. EC25 with 326.28 U/mg (78.25 U/mL), *Daedalea flavida* PE47 with 521.8 U/mg (48.0 U/mL), and finally *Neonothopanus* sp. FE33 with 161.38 U/mg (12.49 U/mL). The laccase activity reported for *Daedalea flavida* PE47 was on day 16. However, this time was not enough to record its maximum activity; since it was observed that in subsequent days, it kept increasing (Figure 3A).

The hydrolysis of casein during the quantification of proteases showed a maximum expression point after 10 days of culture of *Fusarium* sp. FS400 with 160.61 U/mg (88.33 U/mL) followed by *Daedalea flavida* PE47 with 80.67 U/mg (47.24 U/mL) after four days of culture (Figure 3B). For amylases, only two strains of *Fusarium* spp. (PH79 and FS720) from leaf litter and soil, respectively, showed amylase activity after 10 days of culture with 14 U/mg (0.998 U/mL) and 40 U/mg (2.27 U/mL). Regarding *Aspergillus* sp. CH693 and Xylariaceae PH208, under the culture conditions, these strains did not show amylase enzyme activity (Figure 3C).

Xylanase activity was detected in fungi isolated of the three sources, showing the maximum activities at four and six days of culture using *Fusarium* sp. PH79 with 49.23 U/mg (4.41 U/mL) and *Fusarium* sp. FS720 with 36.06 U/mg (3.44 U/mL), followed by *Rigidoporus* sp. FH55 with 24.38 U/mg (216 U/mL) and Xylariaceae PH208 with 20.11 U/mg (2.36 U/mL); only *Neonothopanus* sp. FE33 showed two peaks at 8 and 12 days of culture with 18.82 U/mg (1.88 U/mL) and 19.21 U/mg (1.94 U/mL), respectively (Figure 3D). Regarding lipases, the five strains evaluated *Aspergillus* sp. CH693 with 0.018 U/mg (0.017 U/mL), *Daedalea flavida* PE47 with 0.014 U/mg (0.010 U/mL), *Fusarium* sp. FS400 with 0.045 U/mg (0.015 U/mL), *Marasmius* sp. EC25 with 0.040 U/mg (0.042 U/mL), and *Trametes villosa* FE35 with 0.039 U/mg (0.014 U/mL) showed very low concentrations of lipases during the first 10 days of culture and the activity was lost totally in later days (Figure 3E).

## 4. Discussion

This study analyzes the bioprospecting of five extracellular enzymes produced by fungi isolated from three sources (soil, litter, and wood) collected in an unexplored tropical rainforest. In qualitative tests, the greater number of strains isolated from the soil produce proteases and lipases enzymes; in the leaf litter fungi were proteases, xylanases, and amylases, and in isolates of wood, eight strains had laccase activity and five lipase activity (Table 1 and Figure 1); this suggests specialization of the fungi with the isolation source. A catalytic effect showed the oxidoreductase activity in the solid medium through a dark blue halo around the fungal colonies from the first day of culture, and in the hydrolytic activity, a halo product of hydrolysis was observed from the fourth day of culture (Figure 2). In the case of laccase, guaiacol (0.9 mM), a substrate frequently used to evaluate laccase-producing ligninolytic fungi, was first used [30,44]. However, the affinity constants and the number of rotation of laccases towards guaiacol are low, which can be confused with other oxidases. Therefore, the activity was confirmed with a second test using the ABTS (0.5 mM). This fluorogenic substrate acts as a multi-copper blue oxidase mediator [44,45], excluding isolates that do not produce laccase. As *Neonothopanus* sp. FE33 that in guaiacol test showed a PI 4.6 (data not shown), while in the test with ABTS, the PI was 1.2 (Table 1). Regarding the hydrolytic enzymes, the culture media were the key to detect the presence or absence of the enzyme using only minimal mineral medium and supplemented with the respective substrate as a carbon source [46].

The potency index was recorded as the potential of the halo produced by the strain with a qualitative estimation; however, this PI is not explicitly related to the quantification of the enzyme expressed by the fungus [18,35]. The secretion of several enzymes by the same fungus was vital for the quantitative evaluation phase, i.e., *Neonothopanus* sp. FE33 that showed activity for laccase and xylanase was selected instead of *Panus* sp., which only presented a higher PI for laccase activity (Table 1), even though strains related to the *Panus* genus have been reported to be a good producer of laccase complex enzymes [47,48,49]. Although *Trametes villosa* FE35, *Daedalea flavida* PE47, *Marasmius* sp. CE25, and *Rigidoporus* sp. FH55 collected of wood produce more than one enzyme. These fungal genera with a broad enzymatic spectrum are of great interest since a single strain can hydrolyze substrates of complex composition [50]. 

The results show that the laccase activity of the fungi has a close relationship between the high lignin content and the sources (wood and litter) where they are isolated, expressed mainly by basidiomycete white-rot fungi. These fungi have a unique, versatile, and nonspecific extracellular enzymatic system that allows efficient mineralization of lignin, where laccase enzymes act to obtain their energy source [49,51,52]. Furthermore, many laccase isoenzymes produced by some ligninolytic fungal species may differ between strains, the source of isolation, or the culture medium used in the tests [51].

On the other hand, the laccase activity of *Trametes villosa* (1179 U/mg) is similar to that previously reported in isolates from tropical environments [53,54], demonstrating the efficiency and production in the short term, compared with the activity of isolated fungi in temperate environments [35,55,56]. Additionally, this laccase activity can be improved by adding Cu^2+^ to the culture medium as an inducer [57,58] and/or changing the carbon source [59,60]. Initially, in the quantitative analysis, low laccase activity was obtained, which improved by adding 0.2 mM CuSO_4_; the addition of fructose to the culture medium instead of glucose increased the size and texture of the pellets, the amount of biomass, and the specific activity of the enzyme (data not shown). Possibly a higher N-glycosylation of proteins with fructose oligosaccharides may give the enzyme structure greater stability and protection against degradation caused by proteases during liquid fermentation [50].

It is well understood that macroscopic fungi produce extracellular hydrolytic enzymes [50,61], as *Daedalea flavida* PE47, besides secreting laccases, also produces proteases with values greater than 500 U/mg. Several authors suggest that proteases from basidiomycete fungi isolated from wood are related to nitrogen depletion by breaking down proteins secreted into the environment during secondary metabolism [62,63,64], which leads to protein regulation through physiological processes [65]. Proteases are economically crucial in detergent [66] and food industry [64]; therefore, the production of proteases of *Daedalea flavida* PE47 (80.67 U/mg) could be optimized due to its rapid growth in liquid medium and stability over time (Figure 3B). While *Fusarium* sp. (FS400) presented a protease activity twice that *Daedalea flavida* PE47, and exceeded the values found by other authors for species of the same genus [67,68]. The high production of proteases by the FS400 strain isolated from soil may be due to proteolytic genes expressed by soil microbial communities, which are modulated by substrate’s nutritional and environmental conditions [69]. Furthermore, it has been documented that the proteases of MIF, such as the genus *Fusarium* species, are more efficient due to their ease cultivation, manipulation, growth, stability, and high productivity at low cost [16,70].

The production of xylanases in macroscopic fungi is related to sources rich in xylan, the main component of hemicellulose in leaves and wood [71]. In this case, the isolated strains of wood, *Rigidoporus* sp. FS55 (24.38 U/mg) and *Neonothopanus* sp. FE33 (18.82 U/mg) showed higher xylanase expression, although values were higher than those reported in other studies [72,73]. Whereas, *Fusarium* strains PH79 (49.23 U/mg) from leaf litter and FS720 (36.06 U/mg) from soil presented the best amylase activity, even with values higher than the ones reported in other studies [74,75]. The species of the genus *Fusarium* are strongly associated as phytopathogens; due to their extracellular enzymatic action, they infect the leaves and fruits of plants [76,77]. The importance of amylases and xylanases in agricultural residues, saccharification and fermentation processes is relevant to obtaining bioethanol and its potential in the food industry [39,78]. Therefore, optimization is necessary for a greater expression of the enzyme [79,80].

In the qualitative phase of lipase evaluation, fungi *Marasmius* sp. CE25, *Trametes villosa* FE35, *Daedalea flavida* PE47, *Aspergillus* sp. CH693, and *Fusarium* sp. FS400 showed a high PI to be considered in the quantitative phase. However, under the quantitative experimental conditions, no lipase activity was detected. False positives could cause this due to the interaction with Rhodamine B and the presence of cutinases. The cutinases are members of the α/β hydrolase family, capable of hydrolyzing fatty acid esters and emulsified triacylglycerols with the same efficiency as lipases since they share structural and catalytic characteristics among their molecules [81,82]. Cutinases can be secreted mainly by phytopathogenic fungi from the genera *Fusarium*, *Trichoderma*, *Aspergillus*, *Colletotrichum*, and sometimes by Basidiomycetes [83]; they feed on the cuticle a rigid wall of plants that provides protection, rich in fatty acids of 16 and 18 carbons linked together by ester bonds [84].

The versatility of the humid tropics greatly influences the development and physiology of the fungal species that live there, which is reflected in the catalytic properties of their extracellular enzymes to degrade the sources that they colonize. These enzymatic activities of tropical environmental samples show the importance of assessing and understanding the functioning of fungal biodiversity in these little-explored environments to take advantage of and exploit sustainable compounds [85]. The bioprospecting of fungal organisms isolated from tropical environments is a topic of great interest in biotechnological processes such as biofertilizers, biofuels, bioremediation, biological control, and food processing, detergents, textile, and pharmaceutical industries [86].

## 5. Conclusions

The abundant vegetation and climatic conditions that predominate in tropical rainforests make the development of fungi with particular characteristics possible, such as the catalytic properties and stability of their extracellular enzymes, giving continuity in the recycling of nutrients. Of 150 fungal strains from three sources collected in the humid tropics of southeastern Mexico, 51 strains exhibited laccase, protease, xylanase, amylase, or/and lipase enzymatic activity. A strong relationship between the source of isolation with the expression of the enzyme and its quantification was observed. In the qualitative phase, wood fungi, mostly basidiomycetes, specialize in the production of laccases and lipases; for fungi isolated from leaf litter, expression of amylases, proteases, and xylanases was observed, and in the soil a greater protease and lipase activity. In the qualitative tests, the strains *Trametes villosa* FE35, *Marasmius* sp. CE25, and *Daedalea flavida* PE47 for laccase activity. *Fusarium* spp. PH79 (leaf litter) and FS720 (soil) obtained the highest amylase and xylanase activity yields, respectively. The best protease activity was recorded by *Fusarium* sp. FS400 isolated from soil, and by *Deadela flavida* PE47, the latter reported for the first time its protease activity. Lipase activity was restricted, highlighting the importance of the isolation source. This work represents the first comprehensive study where the enzymatic bioprospecting of fungi isolated from three sources in the humid Mexican tropics is analyzed, with promising results to be applied in future biotechnological, environmental, and industrial studies.

## Figures and Tables

**Table 1:** Primary enzymatic screening of different extracellular enzymes produced by fungal strains isolated from Sierra de Tabasco, Mexico.

Sampling Site	Source	Fungi	Potency Index (PI)				
			Laccase	Protease	Amylase	Xylanase	Lipase
Cocona	Wood	Basidiomycete CE10		2.3			
Cocona	Wood	*Marasmius* sp. CE25 *	1.7				2.3
Cocona	Wood	Basidiomycete CE34		3.0			
La florida	Wood	*Auricularia* sp. FE15					2.5
La florida	Wood	*Neonothopanus* sp. FE33 *	1.2			1.6	
La florida	Wood	*Trametes villosa* FE35 *	2.3				2.1
La florida	Wood	*Hexagonia* sp. FE40		2.0			
La florida	Wood	*Marasmiellus* sp. FE46	1.8				
La florida	Wood	*Rigidoporus* sp. FE55 *	1.9			1.8	
Puyacatengo	Wood	Ascomycete PE37	1.8				
Puyacatengo	Wood	*Marasmius* sp. PE38					2.2
Puyacatengo	Wood	*Panus* sp. PE43	2.1				
Puyacatengo	Wood	*Daedalea flavida* PE47 *	2.2	2.4			2.2
Cocona	Leaf litter	Mycelia sterilia CH464	1.6				
Cocona	Leaf litter	Xylariacea CH240	1.4				
Cocona	Leaf litter	Mycelia sterilia CH546		2.2			
Cocona	Leaf litter	*Monodictys* sp. CH616		2.6			
Cocona	Leaf litter	Mycelia sterilia CH630		2.3			
Cocona	Leaf litter	Mycelia sterilia CH631		2.1			
Cocona	Leaf litter	*Aspergillus* sp. CH665				3.5	
Cocona	Leaf litter	*Drechslera* sp. CH681	1.8				
Cocona	Leaf litter	*Aspergillus* sp. CH693 *			2.5		2.3
La florida	Leaf litter	Mycelia sterilia FH175			2.4		
La florida	Leaf litter	Mycelia sterilia FH321		2.3			
La florida	Leaf litter	Mycelia sterilia FH338			2.2		
La florida	Leaf litter	*Fusarium* sp. FH676					2.3
La florida	Leaf litter	*Fusarium* sp. FH702				3.2	
Puyacatengo	Leaf litter	*Fusarium* sp. PH20		2.2			
Puyacatengo	Leaf litter	*Fusarium* sp. PH79 *			1.9	1.8	
Puyacatengo	Leaf litter	Xylariaceae PH208 *			1.9	1.9	
Puyacatengo	Leaf litter	*Nodulisporium* sp. PH223	1.4				
Puyacatengo	Leaf litter	*Nodulisporium* sp. PH225			1.8		
Puyacatengo	Leaf litter	Mycelia sterilia PH706				2.6	
Cocona	Soil	*Acremonium* sp. CS330				1.2	
Cocona	Soil	Xylariaceae CS708				1.4	
Cocona	Soil	*Aspergillus* sp. CS1022					2.1
Cocona	Soil	*Fusarium* sp. CS1053		2.2			
La florida	Soil	*Fusarium* sp. FS400 *		3			2.1
La florida	Soil	*Paecilomyces* sp. FS446		2.3			
La florida	Soil	*Cylindrocarpon* sp. FS457				1.6	
La florida	Soil	*Paecilomyces* sp. FS470		2.1			
La florida	Soil	*Fusarium* sp. FS720 *			2.5	1.7	
La florida	Soil	*Graphium* sp. FS835					2.3
La florida	Soil	*Fusarium* sp. FS903					2.2
La florida	Soil	Mycelia sterilia FS907					2.2
La florida	Soil	Mycelia sterilia FS943					2.3
Puyacatengo	Soil	*Acremonium* sp. PS595		2.8			
Puyacatengo	Soil	*Aspergillus* sp. PS948		2.7			
Puyacatengo	Soil	Ascomycete PS1130		3.2			
Puyacatengo	Soil	*Graphium* sp. PS1143					2.2
Puyacatengo	Soil	*Gliomastix* sp. PS1298		2.4			

* Fungi selected for the enzymatic activity quantification tests by submerged fermentation.

## Data Availability

Not applicable.
